# Post-sampling mortality and non-response patterns in the English Cancer Patient Experience Survey: Implications for epidemiological studies based on surveys of cancer patients

**DOI:** 10.1016/j.canep.2015.12.010

**Published:** 2016-04

**Authors:** Gary A. Abel, Catherine L. Saunders, Georgios Lyratzopoulos

**Affiliations:** a Cambridge Centre for Health Services Research, Institute of Public Health, University of Cambridge, Forvie Site, Robinson Way, Cambridge CB2 0SR, UK; b RAND Europe, Westbrook Centre, Milton Road, Cambridge CB4 1YG, UK; c Health Behaviour Research Centre, Department of Epidemiology & Public Health, University College London, 1-19 Torrington Place, London WC1 E 6BT, UK

**Keywords:** Patient, Survey, Non-response, Mortality, Cancer, Disparities

## Abstract

**Background:**

Surveys of the experience of cancer patients are increasingly being introduced in different countries and used in cancer epidemiology research. Sampling processes, post-sampling mortality and survey non-response can influence the representativeness of cancer patient surveys.

**Methods:**

We examined predictors of post-sampling mortality and non-response among patients initially included in the sampling frame of the English Cancer Patient Experience Survey. We also compared the respondents’ diagnostic case-mix to other relevant populations of cancer patients, including incident and prevalent cases.

**Results:**

Of 109,477 initially sampled cancer patients, 6273 (5.7%) died between sampling and survey mail-out. Older age and diagnosis of brain, lung and pancreatic cancer were associated with higher risk of post-sampling mortality. The overall response rate was 67% (67,713 respondents), being >70% for the most affluent patients and those diagnosed with colon or breast cancer and <50% for Asian or Black patients, those under 35 and those diagnosed with brain cancer. The diagnostic case-mix of respondents varied substantially from incident or prevalent cancer cases.

**Conclusions:**

Respondents to the English Cancer Patient Experience Survey represent a population of recently treated cancer survivors. Although patient survey data can provide unique insights for improving cancer care quality, features of survey populations need to be acknowledged when analysing and interpreting findings from studies using such data.

## Highlights

• In the context of an English national cancer patient survey we examined predictors of post-sampling mortality or non-response.
• Patients with certain poor prognosis cancers and older patients were substantially more likely to die before survey mail out.
• Response rate was overall high, but was substantially lower in younger, poorer and ethnic minority patients.
• Generalisability limitations need to be acknowledged when analysing and interpreting findings from cancer patient surveys.
• High response rates and short intervals to mail-out can limit concerns about the generalisability of cancer patient survey.

## 1 Introduction

National surveys of the experience of cancer patients are being introduced in different countries. For example, in England national surveys of cancer patients have been conducted initially in 2000 and 2004 and subsequently another four times thus far since 2010, and similar surveys are being planned or have been recently carried out in countries including the US, Norway, Germany and Australia [1], [2], [3], [4]. The primary objective of such surveys is to motivate and inform service improvement efforts, but they also provide new opportunities for research about disparities in the processes and outcomes of cancer care. A large number of such research publications has emerged recently, including research examining disparities in patient experience [5], [6], [7], organisational or geographical differences in hospital performance [8], [9] diagnostic timeliness [10], [11], [12], [13], [14], [15], or other aspects of cancer health services improvement research [16], [17], [18]. An important consideration in appropriately interpreting data from such surveys is the representativeness of respondents which, in the context of cancer patient surveys, may be limited by three factors.

First, if the surveys focus on care experience (as opposed to longer term patient-reported outcomes such as the quality of life of cancer survivors) patients are often sampled following a treatment episode, typically at a hospital, and they are, therefore, unlikely to be representative of either incident or prevalent cancer cases. Second, some of the initially sampled patients will die soon after their treatment and before they could be asked to participate in a survey. This poses specific concerns for cancer compared to general patient surveys, given the relatively high mortality associated with cancer. Third, response rates may vary between different patient groups [19]. Altough socio-demographic characteristics (such as age or socioeconomic status) are known to affect participation in any type of survey, in surveys of cancer patients response rates may be additionally affected by disease severity, with patients with poor prognosis possibly being too ill to respond.

Direct evidence quantifying how sampling processes, post-sampling mortality and survey non-response may influence the representatives of cancer patient surveys is currently lacking, in spite of the increasing availability and use of such data. Against this background, we set out to examine the characteristics of respondents compared to patients included in the sampling frame of the English Cancer Patient Experience Survey, and compare their diagnostic case-mix with that of other relevant populations of cancer patients. In doing so, our aim was to inform how the findings of research based on cancer patient surveys could be best reported, interpreted and contextualised by researchers, policy-makers, clinicians, managers and patient organisations.

## 2 Methods

### 2.1 Data

We analysed data from the sampling frame of the 2010 English Cancer Patient Experience Survey. The survey is carried out by a specialist survey provider (Quality Health) on behalf of the Department of Health. The sampling period was 1st of January to 31st March 2010, and lists of non-deceased patients who received inpatient or day-case cancer treatment in an NHS hospital during that period formed the initial sampling frame (Table 1) [20]. Patients eligible for inclusion were identified by each NHS hospital using the Patient Administration System records. The survey was mailed out at approximately 2–3 months from the end of the sampling period (i.e. late May/June 2010). To minimise the risk of the survey being sent to the former residence of patients who have died soon after their treatment episode, vital status checks (via hospital records or through database checks with the Demographic Batch Service) were conducted during the period from hospital discharge and survey mail-out, and patients found to have died or be otherwise ineligible (e.g. due to changed address) were not sent a survey. Two postal reminders were sent to non-respondents. Anonymous data on patients initially included in the sampling frame were made available for research purposes to the authors by the survey provider.

Information (based on hospital records) on age, sex, ethnicity, cancer diagnosis, and an ecological measure of socioeconomic status (2007 Index of Multiple Deprivation (IMD) of the lower super output area of patients’ residence [21]) was available for patients in the sampling frame. A 36-group classification of cancer diagnoses was used, as in previous analyses of data from this survey, to include patients with both common and rarer cancer [5], [8], [9]. Data were complete for all variables other than ethnicity and deprivation group (which were missing for 8.4% of records in the sampling frame, Supplementary material 3). For analyses exploring predictors of either post-sampling mortality or non-response, only patients with complete covariate information were included. However, for comparisons with other relevant populations of cancer patients, all patients (who were either sampled or responded, as applicable) were included. In line with best practice, non-eligible patients (including those who had died before survey mail out, and those who had moved address) were excluded from the denominator in calculation of response rates (Supplementary material 3) [20], [22].

### 2.2 Analysis

There were three steps in the analysis. First, using multivariable logistic regression (adjusting for age, sex, deprivation, ethnicity and cancer diagnosis) we examined predictors of post-sampling mortality. Here, post-sampling mortality refers to patients who were initially included in the sampling frame and were later known to have died before survey mail out. Noting that when hospitals were aware of deaths (for example in inpatients) the patient was expected to be excluded from the sampling frame. In these models, death is used as an outcome and age-group, sex, deprivation, ethnicity and cancer diagnosis are included as categorical exposure variables. Secondly, we used multivariable logistic regression to examine predictors of non-response among patients who were eligible for inclusion in the denominator (e.g. excluding those known to have died between the creation of the sampling frame and survey mail-out). This model used survey response as the outcome, but otherwise was the same as the above model for mortality. A random effect for hospital of treatment was also included in the above regression models to account for potential clustering of various patient groups within certain hospitals. Thirdly, we compared the diagnostic case-mix of respondents and incident cases; and additionally, for patients with 10 common cancers, we compared the diagnostic case-mix of patients initially included in the sampling frame with that of survey respondents, patients admitted to hospital with a principal diagnosis of cancer, and incident and prevalent cancer cases in the general population, using information from relevant external data sources [23], [24], [25], [26]. We also used the survey question on radiotherapy use to explore how treatment modality may affect survey item non-response [27]. All analyses were carried out using Stata 11.1.

## 3 Results

### 3.1 Predictors of post-sampling mortality after inclusion in the survey sampling frame

**Fig. 1:**
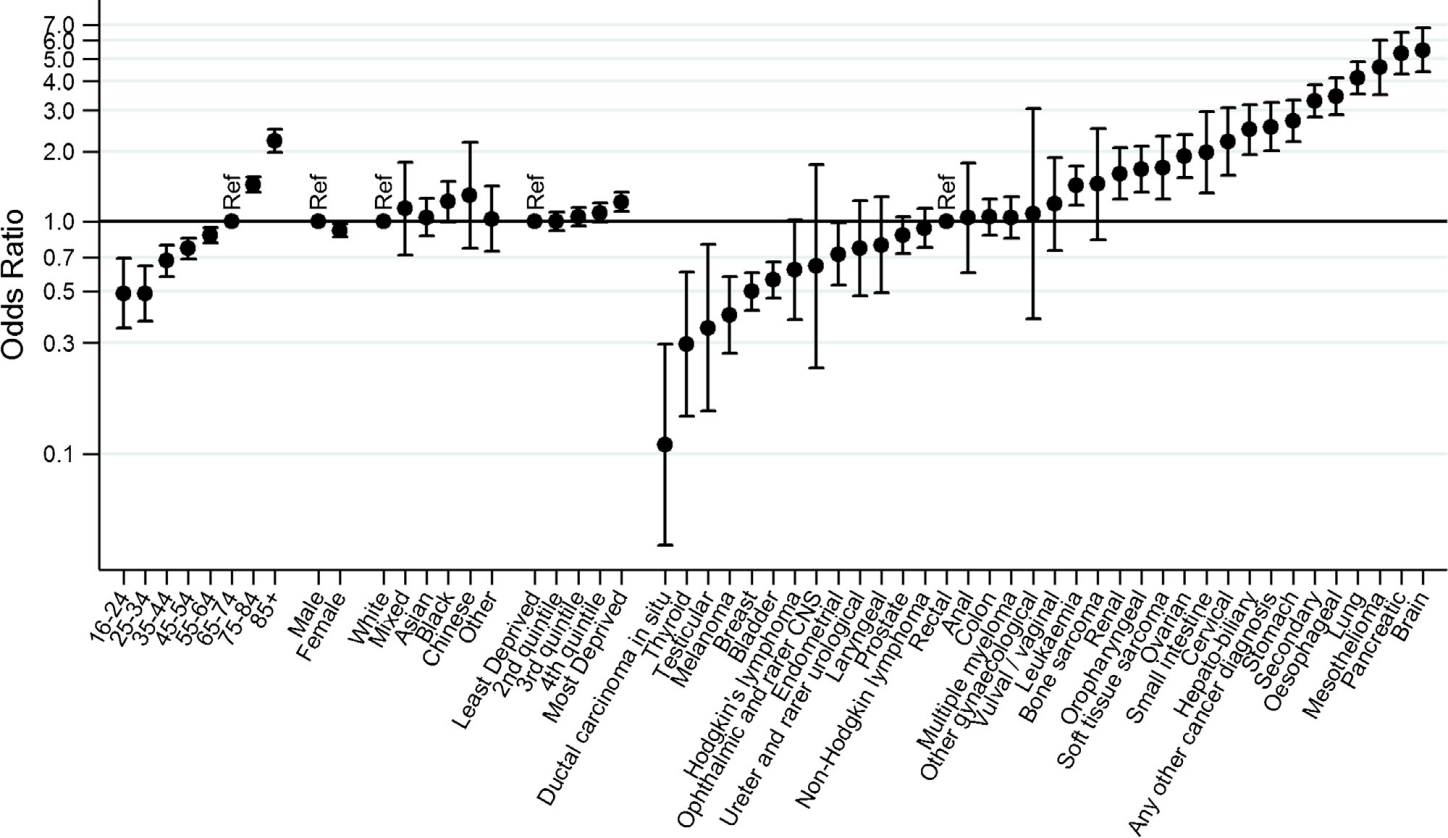
Variation in post-sampling mortality: adjusted odds ratios of post-sampling mortality after initial inclusion in the sampling frame, by socio-demographic characteristic and cancer diagnosis. (*p *< 0.0001 for age and cancer diagnosis, *p* = 0.0047 for sex, *p* = 0.45 for ethnicity, *p* = 0.0002 for deprivation; estimates from multivariable regression, adjusted for all variables shown).

There were 109,475 patients initially included in the sampling frame, of whom 6273 (5.7%) were identified as having died soon after their initial inclusion in the sampling frame. Although the overall degree of sample attrition due to post-sampling mortality was small, there were large relative differences between different patient groups. In crude analysis, exclusion from the survey due to post-sampling mortality was greater for men, older patients, and those from deprived areas (Table 2, Supplementary material 1, *p *< 0.0001 for all). Further, post-sampling mortality was greater than 10% among patients with a diagnosis of pancreatic (19.3%), brain (16.5%), mesothelioma (16.3%), lung (14.9%), oesophageal (13.8%), stomach (11.4%) and hepato-biliary cancer (11.0%). In contrast, post-sampling mortality was lowest among patients with a diagnosis of ductal carcinoma in situ (0.4%), thyroid (1.0%), testicular (1.1%), melanoma (1.8%) and breast cancer (1.8%) (*p *< 0.0001 for variation in post-sampling mortality by cancer). Multivariable logistic regression confirmed very large (>50-fold) variation by cancer diagnosis in the adjusted odds of post-sampling mortality after initial inclusion in the sampling frame. Those with brain cancer, pancreatic cancer and mesothelioma were most likely to have died between sampling and survey mail-out [adjusted odds ratio compared with patients with rectal cancer (95% confidence interval) 5.46 (4.39–6.79), 5.28 (4.31–6.48) and 4.59 (3.51–6.01), respectively]. In contrast, patients with ductal carcinoma in situ, thyroid and testicular cancer diagnoses were least likely to have died between sampling and survey mail-out [adjusted odds ratio compared with patient with rectal cancer of 0.11 (0.04–0.30) 0.09, 0.30 (0.14–0.60) and 0.35 (0.15–0.79), respectively] (Fig. 1, Supplementary material 1, *p *< 0.0001 for variation in post-sampling mortality by cancer).

### 3.2 Predictors of non-response

**Fig. 2:**
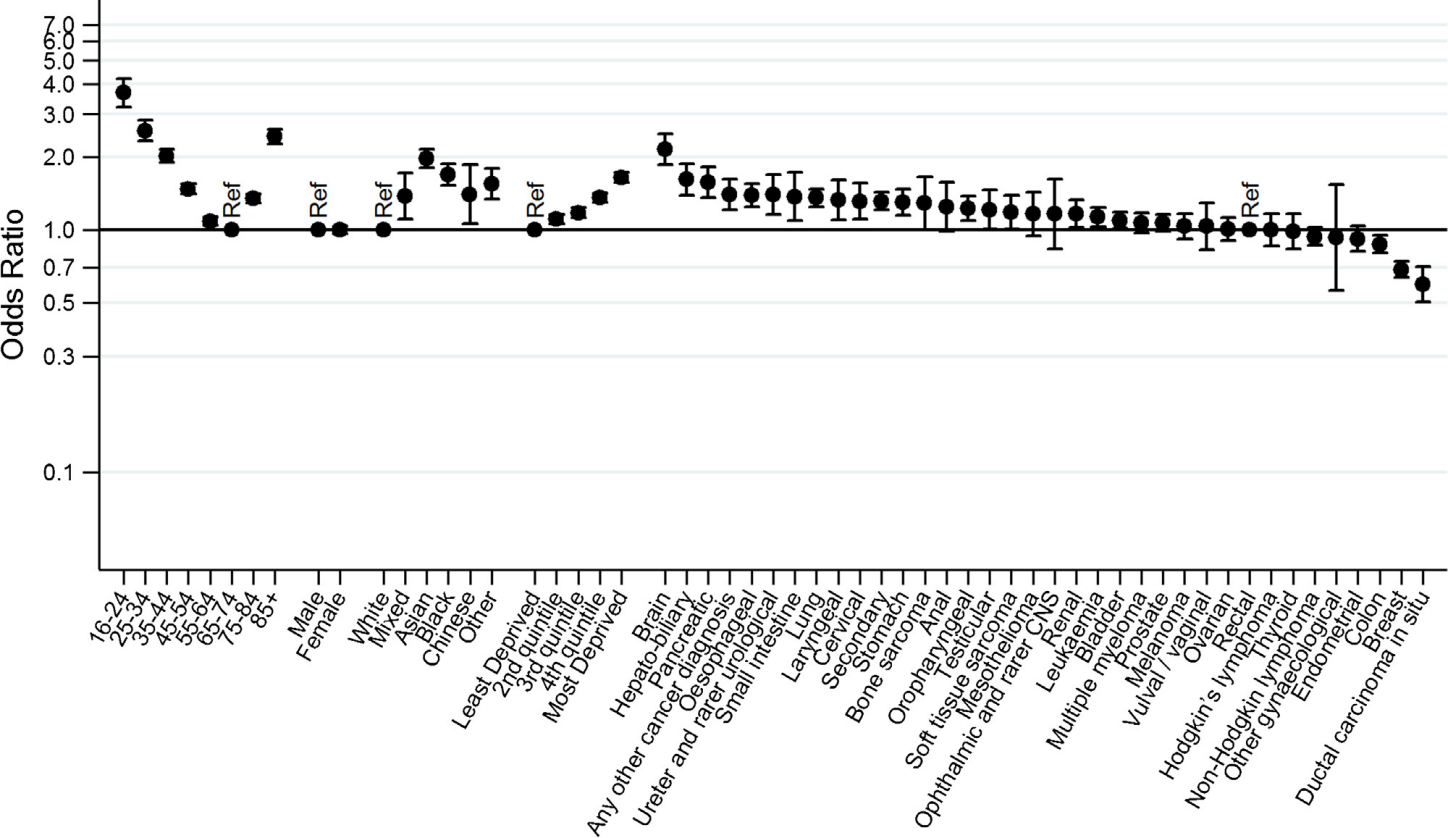
Variation in non-response: adjusted odds ratios for survey non-response by socio-demographic characteristic and cancer diagnosis. (*p *< 0.0001 for all except *p* = 0.90 for sex; estimates from multivariale regession, adjusted for all vaiables shown).

After excluding patients who died between sampling and survey mail-out or were otherwise ineligible, the overall survey response rate was 67% (Supplementary material 3). Response rates varied between different patient groups and were greater than 70% among patients aged from 55 to 74, those living in the most affluent areas and among patients with colon, endometrial, breast cancer or ductal carcinoma in situ. In contrast, the response rate was less than 50% among cancer patients aged under 35, Asian or Black patients, and those with brain cancer (Table 2, Supplementary material 1, *p *< 0.0001 for variation in non-response by age, sex, ethnicity, deprivation and cancer). Multivariable analysis confirmed these patterns of variation in non-response, except for loss of significance in variation by sex (Fig. 2). However, in contrast with the large variation in odds of short-term mortality by cancer diagnosis (see above), there was relatively limited (<4-fold) variation in odds of non-response between patients with different cancers. Moreover, in its greatest part variation in non-response was concentrated in a few cancers, and after excluding ductal carcinoma in situ, breast and brain cancer there was <2-fold variation in the odds of non-response between patients with the other (33) cancer diagnosis groups.

### 3.3 Comparison of cancer diagnosis case mix of surveyed and other relevant cancer patient populations

**Fig. 3:**
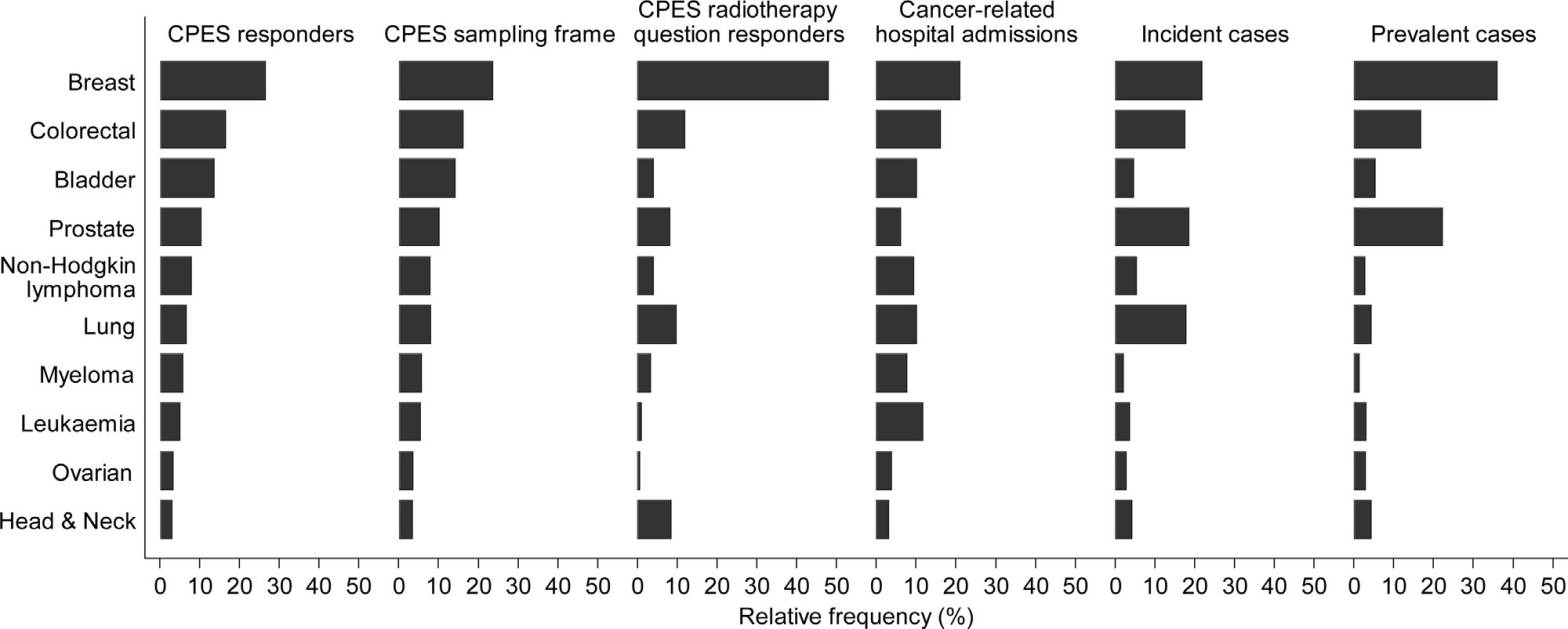
Relative frequency of ten common cancer diagnosis groups across different populations of cancer patients. Note that the diagnostic case-mix of CPES respondents is quite similar to that of cancer-related hospital admissions, but quite dissimilar to that of incident or prevalent cancer cases. (Exact figures and number of patients/cases are given in Supplementary material 2).

There are overall substantial differences in the diagnostic case-mix of survey respondents and incident cases (Supplementary material 4). Specifically, we compared the diagnostic case-mix of survey respondents with other relevant cancer populations for patients with 10 common cancers. Firstly, we note that the diagnostic case-mix of survey respondents (regarding the 10 common cancers) is very similar to that of the sampling frame (Fig. 3, comparing column 1 with 2). Second, the diagnostic case-mix of respondents was broadly similar to that of patients admitted to hospital with a principal diagnosis of cancer, with few exceptions such as for leukaemia and multiple myeloma (Fig. 3, comparing column 1 with 4). Thirdly, the diagnostic case-mix of respondents (and sampled patients) differs substantially from that of either incident or prevalent cases in the general population (Fig. 3, comparing columns 1–2 with 5–6). For example, patients with bladder cancer are over-represented among survey respondents and sampled patients, compared with both incident and prevalent cases. Finally, we note that the diagnostic case-mix of survey respondents who replied to questions specific to certain treatment modalities may be very different to that of respondents and sampled patients, as exemplified by respondents treated by radiotherapy (Fig. 3, comparing columns 1–2 with 3).

## 4 Discussion

### 4.1 Main findings

Respondents to the English Cancer Patient Experience Survey represent a population of cancer survivors who have recently received hospital treatment for their cancer. Consequently, the diagnostic case-mix of respondents varies substantially from both that of incident and prevalent cases in the general population. After inclusion in the sampling frame, older and lower socioeconomic status patients and those with poor prognosis cancers experience a higher risk of post-sampling mortality during the short (2–3 month) period from their treatment and survey mail out. Among patients who could provide a response, non-respondents are more likely to be young, non-White and socioeconomically deprived, with no difference by sex. Although both post-sampling mortality and response rate vary by cancer, this variation has relatively little impact on the diagnostic case-mix of respondents with 10 common cancers compared to the sampling frame.

### 4.2 Findings in the context of previous work; what is known and what this study adds

The observed patterns of variation in post-sampling mortality by cancer reflect general patterns of variation in survival for different, good, average and poor prognosis cancers [28]. There were notable differences in post-sampling mortality (i.e. between sampling frame creation and survey mail-out) by deprivation group, which seem to reflect known socioeconomic inequalities in cancer survival [29]. These observations illustrate the potential for differential patterns of post-sampling mortality to affect the representativeness of cancer patient surveys, a concern that is particularly applicable to surveys of cancer patients because of the relatively high mortality associated with cancer. Nonetheless, in the context of the Cancer Patient Experience Survey there is a relatively short interval between treatment and mail-out, which minimises the effect of differential post-sampling mortality on the diagnosis case-mix of the cancers of included patients. Cancer patient surveys with longer intervals between sample definition and mail out will be more prone to case-mix distortion due to post-sampling mortality.

The English Cancer Patient Experience Survey has a relatively high response rate compared to other patient experience surveys. For example the US Hospital Consumer Assessment of Healthcare Providers and Systems survey, the English General Practice Patient Survey and the English Adult Inpatient Survey have typical response rates between 30% and 50% [30], [31], [32]. However, in spite of a high response rate, we identified large variation in response rates between different patient groups. The findings that younger and more deprived patients and those from ethnic minorities are less likely to respond to patient experience surveys are consistent with previous work, but we observed small only differences in response rates by sex [19]. Variation in response rates by cancer was relatively small, compared with variation by cancer in post-sampling mortality (indeed age appears to be a more important independent predictor of non-response than cancer diagnosis—see Fig. 2 which presents the adjusted odds of non-response by age, sex, ethnicity, deprivation and cancer diagnosis). For this reason, and in the context of a high overall response rate, there were only minor differences in the diagnostic case-mix of common cancers between respondents and sampled patients.

The diagnostic case-mix of survey respondents is similar (although not identical) to that observed among patients with a hospital admission with a principal diagnosis of cancer, but where differences are noted, they may reflect variation in the need for multiple treatment episodes, and their frequency between patients with different cancers. For example, the relative proportion of patients with a diagnosis of leukaemia or multiple myeloma is lower among respondents than among patients with hospital admission for cancer. Some of these patients will have more than one chemotherapy treatment session during the sampling period (therefore they will be over-represented among the population of patients who are admitted to hospital for cancer) but will only be sampled once and be sent a single questionnaire. In contrast, the diagnostic case-mix of survey respondents is dissimilar to that of either incident or prevalent cancer cases. Again these differences are likely to reflect variation in treatment patterns for different cancers. This is exemplified by patients with bladder cancer, many of whom will have follow-up cystoscopies at regular intervals for a long period after diagnosis as part of their management, and who are for this reason over-represented among respondents (and sampled patients), compared with broader populations of incident or prevalent cancer cases. The converse pattern is apparent for patients with prostate cancer: relatively few such patients would receive hospital-based treatments such as surgery or radiotherapy, and for this reason they are under-represented among respondents (and sampled patients) compared with incident and prevalent cases.

### 4.3 Strengths and limitations

Our study describes the representativeness of a large national survey of cancer patients examining post-sampling mortality and non-response patterns. Unlike most of the evidence on predictors of non-response, in patient surveys in general, we were able to examine these phenomena using information on cancer type (diagnosis group) in addition to socio-demographic variables. Hospital records are known to contain degrees of inaccuracy (e.g. regarding the assignment of ethnicity [33], or diagnosis) but such errors could not possibly account for the full size of the very substantial variations in either post-sampling mortality or non-response that we observed.

The ascertainment of post-sampling mortality is restricted to deaths identified between sampling frame creation and survey mail-out. If hospitals were aware that treated patients died at the point of sampling frame creation these patients would have been excluded. Consequently, the findings will underestimate the overall short-term mortality after hospital treatment for cancer and may also under-estimate the size of respective variation by patient group. However, the diagnostic case-mix of cancer-related hospital admissions and that of patients included in the initial sampling frame are very similar for common cancers, suggesting that the potential for under-ascertainment of overall short-term (i.e. including inpatient as well as post-sampling) mortality after cancer treatment is likely to be small.

In focusing on sample characteristics and non-response patterns we are not suggesting that these are the only methodological issues worthy of consideration when considering the use of data from surveys of cancer patients for purpose of descriptive epidemiology. For example, cognitive validation of survey items is also important.

### 4.4 Implications

Cancer policy makers and users of findings from cancer patient experience surveys need to be aware of the characteristics of respondents. As we have shown, in postal surveys of recently treated cancer patients, respondents are likely to be representative of cancer survivors who recently received hospital treatment. However, this may not be true if the interval between sampling period and survey mail-out is substantial [34]. Because the make-up of incident, prevalent, and recently treated cases are necessarily different, no single sampling strategy can provide a selection of patients that is representative of each one of these populations of cancer patients.

The implications discussed below are specific to surveys which sample cancer patients on the basis of recent treatment such as is the case, for example, with recent studies in England, Germany and Norway [2], [3], [12], [13]. Surveys which sample different populations (for example a survey of incident cases in the USA) will have parallel issues when ascertaining inferences about different populations [1]. Although our discussion is focusing on surveys of cancer patients, similar concerns may also apply in context of surveys of patients with other conditions where mortality, treatment modality and sample definitions affect the representativeness of survey results.

Caution is needed in interpreting data from surveys of cancer patients when they are used to study the care of incident or prevalent cases (e.g. when studying outcomes relating to processes of cancer care up to and including diagnosis, or care management in the community after hospital treatment, respectively). Crude estimates of outcome prevalence in survey respondents are bound to be biased compared to the true value in the relevant population (e.g. incident or prevalent cases). Partial improvement upon such biased estimates can be achieved by weighting to account for compositional differences between survey respondents and the population of relevance [22]. However, such approaches will not obviate potential for selection bias within strata of the weighting variables. For example, selection bias which can be introduced through differential mortality in otherwise similar respondents (in terms of age, sex, cancer diagnosis etc.) who nonetheless have differential prognosis.

When data from patient surveys are used to measure hospital performance recently treated cancer survivors are indeed the population of prior interest, and this minimises concerns about generalisability in this context. However, there may still be concerns about case-mix distortion due to post-sampling mortality and non-response. Non-response weighting could be applied to any estimates of prevalence of patient reported outcomes or hospital scores, as is standard practice in some other surveys (e.g. the English General Practice Patient Survey). An alternative approach, which we recommend, is to use case-mix adjusted estimates of hospital performance. When making comparisons of hospital performance such estimates would account for the variable diagnostic and demographic mix of patients treated by different hospitals; further, under certain assumptions they will also account for variation in post-sampling mortality and non-response between hopsitals [9]. Similarly, when the objective of the analysis is to estimate associations (as is the case in research aiming to identify disparities in care experience) case-mix adjustment obviates concerns about potential non-response bias. While acknowledging different viewpoints about the use of case-mix adjustment in patient-reported outcomes and disparities research, in general we advocate public reporting of both crude and case-mix adjusted estimates of hospital performance of cancer patient experience [9], [35].

Previous work has identified that younger patients and those from ethnic minorities report poorer experiences of cancer care [5], [7], [9], as do patients with advanced stage cancer (and consequently poor prognosis) [36]. These are the same patient groups who are likely to be under-represented in survey respondents—a ‘double whammy’ of both survey under-representation and inequality in experience. We recommend that existing surveys can be re-designed (or additional surveys designed anew) so that patients are invited to participate shortly after (or before) discharge from hospital care. Doing so should be expected to increase representation of patient groups with poorer prognosis (a concern also highlighted by advocacy organisations representing such patients [37]), and might also help to increase response rates. Certainly long intervals between treatment and survey should be avoided.

## 5 Conclusions

The case-mix of respondents to surveys of cancer patients is determined largely by the way that the sample is defined. There are a number of relevant populations that can be defined and no survey will represent all of them. As is the case with the English Cancer Patient Experience Survey, respondents will differ from incident or prevalent cases in the general population if they are recruited on the basis of recent hospital treatment. Survey respondents will also differ from the patients initially included in the sampling frame of these surveys, because of both differential risk of post-sampling mortality, and differential non-response, although high response rates and short intervals between treatment and survey mail-out limit such concerns. These issues need to be borne in mind when interpreting and using data from such surveys. If however the experience of certain patient groups (e.g. of patients with poor prognosis cancers) is of prime prior interest, alternative survey designs need to be considered.

## Authorship contribution

The study was originally conceived by GL, but research questions and methods employed to answer them were subsequently substantially enriched by both CLS and GAA. Methods development, data interpretation and writing were done collaboratively by all authors (CLS, GAA, GL). The principal analyst was CLS.

## Conflicts of interest

None.

## Figures and Tables

**Table 1:** Survey phases and timeline: English Cancer Patient Survey 2010. Please note that other than for the sampling period (Jan–Mar 2010), timings are approximate and may have varied for individual hospitals.

January–March 2010	April–June 2010	June 2010	2–3 month period from June 2010
Patients are treated for cancer at an English National Health Service (NHS) hospital	Eligible (non-deceased) patients are identified by hospitals and initial patient lists are sent to the survey provider	Survey mail out	Completed surveys returned to survey provider Mail out of up to two reminders to initial non-respondents
Duplicate patients (e.g. treated in more than one hospital) are identified and database and hospital record checks are made for patient deaths


**Table 2:** Patient characteristics and cancer diagnoses of respondents to the 2010 Cancer Patient Experience Survey (CPES) and associations with post-sampling mortality and response rates.^a^

	Sampling frame	Post-sampling deaths (*N*,%)		Respondents	Response rate^b^		Sampling frame	Post-sampling deaths (*N*,%)		Respondents	Response rate^b^
Age						Cancer					
16–24	1,149	38	3.3	408	37.9	Ductal carcinoma in situ	955	4	0.4	732	77.8
25–34	2,212	61	2.8	992	47.4	Thyroid	780	8	1.0	458	61.3
35–44	5,983	205	3.4	3,239	57.0	Testicular	567	6	1.1	275	50.4
45–54	13,208	547	4.1	8,091	64.7	Melanoma	1,808	32	1.8	1,146	65.9
55–64	24,245	1,257	5.2	16,066	70.6	Breast	16,937	302	1.8	12,204	73.8
65–74	29,172	1,744	6.0	19,443	71.8	Bladder	10,544	311	2.9	6,591	65.4
75–84	19,468	1,488	7.6	11,570	65.4	Hodgkin’s lymphoma	946	18	1.9	509	55.3
85+	4,797	482	10.0	2,172	51.7	Ophthalmic and rarer CNS	194	4	2.1	108	63.2
						Endometrial	1,878	55	2.9	1,269	70.3
Gender						Ureter and rarer urological	544	20	3.7	313	60.2
Men	48,497	3,179	6.6	29,067	65.1	Laryngeal	550	20	3.6	319	61.3
Women	51,737	2,643	5.1	32,914	67.9	Prostate	7,343	302	4.1	4,710	68.2
						Non-Hodgkin lymphoma	5,805	225	3.9	3,768	68.6
Ethnicity						Rectal	4,923	213	4.3	3,187	68.4
White	94,447	5,472	5.8	59,382	67.6	Anal	355	15	4.2	208	62.3
Mixed	376	21	5.6	188	54.2	Colon	6,874	319	4.6	4,619	71.2
Asian	2,386	145	6.1	1,022	46.2	Multiple myeloma	4,098	189	4.6	2,569	66.5
Black	1,933	124	6.4	857	48.5	Gynaecological NOS	79	4	5.1	51	68.0
Chinese	228	17	7.5	118	56.2	Vulval/vaginal	417	22	5.3	253	65.7
Other	864	43	5.0	414	52.0	Leukaemia	4,144	233	5.6	2,375	61.8
						Bone sarcoma	320	15	4.7	156	51.8
Deprivation^c^						Renal	1,422	92	6.5	843	64.6
Most affluent	20,809	1,079	5.2	13,932	71.3	Oropharyngeal	2,016	132	6.5	1,147	62.0
2	21,655	1,145	5.3	14,093	69.6	Soft tissue sarcoma	873	58	6.6	494	62.6
3	20,803	1,175	5.6	13,197	68.0	Ovarian	2,620	185	7.1	1,627	68.0
4	19,369	1,167	6.0	11,455	63.8	Small-intestine	382	30	7.9	210	60.9
Least affluent	17,598	1,256	7.1	9,304	58.1	Cervical	729	48	6.6	359	53.4
						Hepato-biliary	993	109	11.0	488	56.0
						Any other cancer diagnosis	1,166	119	10.2	599	59.2
						Stomach	1,749	199	11.4	936	61.7
						Secondary	6,974	803	11.5	3,836	62.8
						Oesophageal	2,457	339	13.8	1,280	61.5
						Lung	5,873	878	14.9	3,050	61.8
						Mesothelioma	565	92	16.3	315	67.3
						Pancreatic	1,199	231	19.3	555	58.4
						Brain	1,155	190	16.5	422	44.7

a The sample described here are the 100,234 patients with complete hospital record ethnicity, and deprivation information.

b Calculated as the proportion of people in each group who responded to the survey after excluding ineligible patients (those who had died, and other ineligible patients).

c Measured using the index of multiple deprivation, and quantile-defining cut points 8.257, 13.525, 20.741, and 33.511.
