# Supplementary material for: Post-sampling mortality and non-response patterns in the English Cancer Patient Experience Survey: Implications for epidemiological studies based on surveys of cancer patients
Source: Cancer Epidemiol. 2016 Apr;41:34–41. doi: 10.1016/j.canep.2015.12.010 (PMC4819677; doi:10.1016/j.canep.2015.12.010)
Supplement: Supplementary file 1 [file mmc1.docx]

**Supplementary material 1. Crude and adjusted odds ratios for post-sampling mortality (A) and survey non-response (B)**

| **A.** | **Post-sampling mortality** | | | |  |
| --- | --- | --- | --- | --- | --- |
| **Age** | **Crude** | | **Adjusted** | |  |
| 16-24 | 0.46 (0.33 - 0.65) | p<0.0001 | 0.49 (0.35 - 0.69) | p<0.0001 |  |
| 25-34 | 0.41 (0.31 - 0.53) |  | 0.49 (0.37 - 0.64) |  |  |
| 35-44 | 0.55 (0.47 - 0.63) |  | 0.68 (0.58 - 0.79) |  |  |
| 45-54 | 0.66 (0.60 - 0.73) |  | 0.76 (0.69 - 0.85) |  |  |
| 55-64 | 0.84 (0.78 - 0.91) |  | 0.87 (0.81 - 0.94) |  |  |
| 65-74 | reference |  | reference |  |  |
| 75-84 | 1.32 (1.23 - 1.42) |  | 1.44 (1.33 - 1.55) |  |  |
| 85+ | 1.80 (1.62 - 2.00) |  | 2.22 (1.98 - 2.48) |  |  |
| **Gender** |  |  |  |  |  |
| Men | reference | p<0.0001 | reference | p=0.0047 |  |
| Women | 0.77 (0.73 - 0.81) |  | 0.91 (0.86 - 0.97) |  |  |
| **Ethnicity** |  |  |  |  |  |
| White | reference | p=0.57 | reference | p=0.45 |  |
| Mixed | 0.88 (0.56 - 1.37) |  | 1.13 (0.71 - 1.80) |  |  |
| Asian | 0.87 (0.73 - 1.04) |  | 1.04 (0.87 - 1.25) |  |  |
| Black | 1.05 (0.86 - 1.27) |  | 1.21 (0.99 - 1.48) |  |  |
| Chinese | 1.19 (0.72 - 1.97) |  | 1.29 (0.77 - 2.18) |  |  |
| Other | 0.89 (0.65 - 1.22) |  | 1.02 (0.74 - 1.41) |  |  |
| **Deprivation** |  |  |  |  |  |
| Most Affluent | reference | p<0.0001 | reference | p=0.0002 |  |
| 2 | 1.02 (0.93 - 1.11) |  | 1.00 (0.91 - 1.09) |  |  |
| 3 | 1.06 (0.97 - 1.16) |  | 1.04 (0.95 - 1.14) |  |  |
| 4 | 1.13 (1.03 - 1.24) |  | 1.09 (0.99 - 1.20) |  |  |
| Least Affluent | 1.29 (1.17 - 1.41) |  | 1.21 (1.10 - 1.33) |  |  |
| **Cancer*** |  |  |  |  |  |
| Ductal carcinoma in situ | 0.09 (0.03 - 0.24) |  | 0.11 (0.04 - 0.30) |  |  |
| Thyroid | 0.23 (0.11 - 0.47) |  | 0.30 (0.14 - 0.60) |  |  |
| Testicular | 0.22 (0.09 - 0.49) |  | 0.35 (0.15 - 0.79) |  |  |
| Melanoma | 0.37 (0.25 - 0.54) | p<0.0001 | 0.39 (0.27 - 0.58) | p<0.0001 |  |
| Breast | 0.40 (0.33 - 0.48) |  | 0.50 (0.41 - 0.60) |  |  |
| Hodgkin's lymphoma | 0.40 (0.25 - 0.66) |  | 0.62 (0.38 - 1.01) |  |  |
| Ophthalmic and rarer CNS | 0.54 (0.20 - 1.48) |  | 0.64 (0.23 - 1.76) |  |  |
| Endometrial | 0.66 (0.49 - 0.89) |  | 0.72 (0.53 - 0.98) |  |  |
| Ureter and rarer urological | 0.85 (0.53 - 1.36) |  | 0.76 (0.48 - 1.22) |  |  |
| Laryngeal | 0.80 (0.50 - 1.28) |  | 0.79 (0.49 - 1.27) |  |  |
| Prostate | 0.96 (0.80 - 1.15) |  | 0.87 (0.72 - 1.04) |  |  |
| Non-Hodgkin lymphoma | 0.87 (0.72 - 1.05) |  | 0.93 (0.77 - 1.13) |  |  |
| Rectal | reference |  | reference |  |  |
| Anal | 0.99 (0.58 - 1.69) |  | 1.03 (0.60 - 1.77) |  |  |
| Colon | 1.08 (0.90 - 1.29) |  | 1.04 (0.87 - 1.25) |  |  |
| Multiple myeloma | 1.05 (0.86 - 1.29) |  | 1.04 (0.85 - 1.27) |  |  |
| Other gynaecological | 1.05 (0.38 - 2.95) |  | 1.08 (0.38 - 3.04) |  |  |
| Vulval / vaginal | 1.22 (0.78 - 1.93) |  | 1.19 (0.75 - 1.88) |  |  |
| Leukaemia | 1.30 (1.07 - 1.57) |  | 1.42 (1.17 - 1.73) |  |  |
| Bone sarcoma | 1.01 (0.59 - 1.75) |  | 1.45 (0.83 - 2.51) |  |  |
| Renal | 1.52 (1.18 - 1.97) |  | 1.60 (1.24 - 2.07) |  |  |
| Oropharyngeal | 1.54 (1.23 - 1.93) |  | 1.67 (1.33 - 2.10) |  |  |
| Soft tissue sarcoma | 1.55 (1.14 - 2.11) |  | 1.70 (1.24 - 2.31) |  |  |
| Ovarian | 1.65 (1.34 - 2.02) |  | 1.90 (1.54 - 2.35) |  |  |
| Small-intestine | 1.86 (1.25 - 2.79) |  | 1.98 (1.32 - 2.96) |  |  |
| Cervical | 1.56 (1.13 - 2.17) |  | 2.20 (1.57 - 3.08) |  |  |
| Hepato-biliary | 2.48 (1.94 - 3.18) |  | 2.48 (1.93 - 3.18) |  |  |
| Any other cancer diagnosis | 2.44 (1.93 - 3.10) |  | 2.55 (2.01 - 3.24) |  |  |
| Stomach | 2.91 (2.37 - 3.57) |  | 2.71 (2.21 - 3.33) |  |  |
| Secondary | 3.04 (2.60 - 3.57) |  | 3.30 (2.81 - 3.87) |  |  |
| Oesophageal | 3.54 (2.95 - 4.24) |  | 3.45 (2.88 - 4.14) |  |  |
| Lung | 4.06 (3.47 - 4.75) |  | 4.13 (3.53 - 4.84) |  |  |
| Mesothelioma | 4.61 (3.53 - 6.01) |  | 4.59 (3.51 - 6.01) |  |  |
| Pancreatic | 5.12 (4.18 - 6.26) |  | 5.28 (4.31 - 6.48) |  |  |
| Brain | 4.11 (3.32 - 5.09) |  | 5.46 (4.39 - 6.79) |  |  |

| **B.** | **Survey non-response** | | | |
| --- | --- | --- | --- | --- |
| **Age** | **Crude** | | **Adjusted** | |
| 16-24 | 4.04 (3.55 - 4.58) | p<0.0001 | 3.67 (3.20 - 4.20) | p<0.0001 |
| 25-34 | 2.70 (2.46 - 2.95) |  | 2.56 (2.33 - 2.82) |  |
| 35-44 | 1.88 (1.77 - 1.99) |  | 2.02 (1.89 - 2.15) |  |
| 45-54 | 1.37 (1.31 - 1.43) |  | 1.47 (1.40 - 1.55) |  |
| 55-64 | 1.06 (1.02 - 1.10) |  | 1.09 (1.04 - 1.13) |  |
| 65-74 | reference |  | reference |  |
| 75-84 | 1.35 (1.30 - 1.41) |  | 1.35 (1.29 - 1.40) |  |
| 85+ | 2.38 (2.23 - 2.55) |  | 2.42 (2.26 - 2.59) |  |
| **Gender** |  |  |  |  |
| Men | reference | p<0.0001 | reference | p=0.90 |
| Women | 0.88 (0.86 - 0.91) |  | 1.00 (0.96 - 1.03) |  |
| **Ethnicity** |  |  |  |  |
| White | reference | p<0.0001 | reference | p<0.0001 |
| Mixed | 1.61 (1.30 - 2.00) |  | 1.38 (1.11 - 1.72) |  |
| Asian | 2.17 (1.99 - 2.37) |  | 1.96 (1.80 - 2.15) |  |
| Black | 1.90 (1.73 - 2.10) |  | 1.69 (1.52 - 1.87) |  |
| Chinese | 1.50 (1.14 - 1.98) |  | 1.40 (1.05 - 1.85) |  |
| **Deprivation** |  |  |  |  |
| Most Affluent | reference | p<0.0001 | reference | p<0.0001 |
| 2 | 1.12 (1.08 - 1.17) |  | 1.11 (1.06 - 1.16) |  |
| 3 | 1.21 (1.15 - 1.26) |  | 1.18 (1.12 - 1.23) |  |
| 4 | 1.43 (1.37 - 1.50) |  | 1.36 (1.29 - 1.42) |  |
| Least Affluent | 1.80 (1.71 - 1.88) |  | 1.64 (1.57 - 1.73) |  |
| **Cancer*** |  |  |  |  |
| Brain | 2.66 (2.30 - 3.07) |  | 2.14 (1.85 - 2.49) |  |
| Hepato-biliary | 1.62 (1.40 - 1.88) |  | 1.61 (1.38 - 1.87) |  |
| Pancreatic | 1.54 (1.33 - 1.78) |  | 1.57 (1.35 - 1.81) |  |
| Any other cancer diagnosis | 1.49 (1.29 - 1.71) |  | 1.40 (1.21 - 1.61) |  |
| Oesophageal | 1.37 (1.23 - 1.52) |  | 1.38 (1.24 - 1.54) |  |
| Ureter and rarer urological | 1.42 (1.18 - 1.72) |  | 1.40 (1.15 - 1.69) |  |
| Small-intestine | 1.40 (1.12 - 1.76) | p<0.0001 | 1.37 (1.09 - 1.72) | p<0.0001 |
| Lung | 1.33 (1.22 - 1.45) |  | 1.35 (1.24 - 1.47) |  |
| Laryngeal | 1.35 (1.12 - 1.64) |  | 1.32 (1.10 - 1.60) |  |
| Cervical | 1.89 (1.60 - 2.22) |  | 1.31 (1.10 - 1.55) |  |
| Secondary | 1.32 (1.22 - 1.43) |  | 1.31 (1.21 - 1.42) |  |
| Stomach | 1.35 (1.19 - 1.52) |  | 1.30 (1.15 - 1.47) |  |
| Bone sarcoma | 1.95 (1.54 - 2.48) |  | 1.28 (1.00 - 1.65) |  |
| Anal | 1.31 (1.04 - 1.66) |  | 1.24 (0.98 - 1.57) |  |
| Oropharyngeal | 1.34 (1.19 - 1.50) |  | 1.23 (1.09 - 1.38) |  |
| Testicular | 2.12 (1.77 - 2.54) |  | 1.21 (1.00 - 1.46) |  |
| Soft tissue sarcoma | 1.30 (1.11 - 1.52) |  | 1.18 (1.00 - 1.39) |  |
| Mesothelioma | 1.06 (0.86 - 1.30) |  | 1.16 (0.95 - 1.43) |  |
| Ophthalmic and rarer CNS | 1.35 (0.98 - 1.86) |  | 1.16 (0.83 - 1.61) |  |
| Renal | 1.18 (1.04 - 1.35) |  | 1.16 (1.02 - 1.33) |  |
| Leukaemia | 1.30 (1.18 - 1.42) |  | 1.13 (1.03 - 1.24) |  |
| Bladder | 1.15 (1.06 - 1.24) |  | 1.09 (1.01 - 1.18) |  |
| Multiple myeloma | 1.06 (0.97 - 1.16) |  | 1.07 (0.97 - 1.17) |  |
| Prostate | 1.03 (0.95 - 1.11) |  | 1.07 (0.98 - 1.16) |  |
| Melanoma | 1.15 (1.02 - 1.29) |  | 1.03 (0.92 - 1.17) |  |
| Vulval / vaginal | 1.15 (0.92 - 1.43) |  | 1.03 (0.82 - 1.29) |  |
| Ovarian | 1.03 (0.93 - 1.15) |  | 1.01 (0.90 - 1.12) |  |
| Rectal | reference |  | reference |  |
| Hodgkin's lymphoma | 1.70 (1.47 - 1.97) |  | 1.00 (0.86 - 1.16) |  |
| Thyroid | 1.36 (1.16 - 1.60) |  | 0.99 (0.83 - 1.16) |  |
| Non-Hodgkin lymphoma | 0.99 (0.91 - 1.07) |  | 0.94 (0.86 - 1.02) |  |
| Other gynaecological | 1.01 (0.62 - 1.65) |  | 0.93 (0.56 - 1.53) |  |
| Endometrial | 0.91 (0.81 - 1.02) |  | 0.92 (0.81 - 1.04) |  |
| Colon | 0.88 (0.81 - 0.96) |  | 0.87 (0.80 - 0.95) |  |
| Breast | 0.77 (0.71 - 0.82) |  | 0.69 (0.64 - 0.74) |  |
| Ductal carcinoma in situ | 0.62 (0.53 - 0.74) |  | 0.59 (0.50 - 0.71) |  |

*Cancer diagnosis classification by ICD-10 diagnostic code for this figure as for figure 3 (ordered by adjusted odds ratio for post-sampling mortality) Ductal carcinoma in situ D05; Thyroid C73; Testicular C62; Melanoma C43; Breast C50; Bladder C67; Hodgkin lymphoma C81; Ophthalmic and rarer CNS C47, C69, C70, C72; Endometrial C54, C55; Ureter and rarer urological C60, C63, C65, C66, C68; Laryngeal C32; Prostate C61; Non-Hodgkin lymphoma C82, C83, C85, C84; Rectal C19, C20; Anal C21; Colon C18; Multiple myeloma C90; Other gynaecological C57; Vulval / vaginal C51, C52; Leukaemia C91, C92, C93, C94, C95; Bone sarcoma C40, C41; Renal C64; Oropharyngeal C00 - C14, C30, C31; Soft tissue sarcoma C48, C49, C46; Ovarian C56; Small intestine C17, C26; Cervical C53; Hepato-biliary C22, C23, C24; Any other cancer diagnosis C37, C38, C39, C74, C75, C76, C80, C97, C58, C88, C96; Stomach C16; Secondary C77, C78, C79; Oesophageal C15; Lung C34, C33; Mesothelioma C45; Pancreatic C25; Brain C71

**Supplementary material 2. Percentages underlyling Figure 3 (Relative frequency of ten common cancer diagnosis groups across different populations of cancer patients)**

|  | **CPES responders**  **n (%)** | **CPES sampling frame**  **n (%)** | **CPES radiotherapy question responders**  **n (%)** | **Cancer-related hospital admissions**  **n (%)** | **Incident cases**  **n (%)** | **Prevalent cases**  **n (%)** |
| --- | --- | --- | --- | --- | --- | --- |
| Breast | 13,444 (26.6) | 18,673 (23.8) | 5,432 (48.0) | 173,815 (21.1) | 40,585 (21.9) | 250,297 (36.0) |
| Colorectal | 8,447 (16.7) | 12,763 (16.3) | 1,358 (12.0) | 133,705 (16.2) | 32,751 (17.7) | 117,434 (16.9) |
| Bladder | 6,970 (13.8) | 11,147 (14.2) | 466 (4.1) | 84,584 (10.2) | 8,946 (4.8) | 37,977 (5.5) |
| Prostate | 5,270 (10.4) | 8,175 (10.4) | 932 (8.2) | 51,048 (6.2) | 34,593 (18.7) | 154,585 (22.3) |
| Non-Hodgkin lymphoma | 4,055 (8.0) | 6,267 (8.0) | 450 (4.0) | 78,426 (9.5) | 10,217 (5.5) | 20,204 (2.9) |
| Lung | 3,361 (6.7) | 6,455 (8.2) | 1,122 (9.9) | 84,321 (10.2) | 33,150 (17.9) | 30,259 (4.4) |
| Myeloma | 2,936 (5.8) | 4,659 (5.9) | 382 (3.4) | 64,661 (7.8) | 4,235 (2.3) | 10,180 (1.5) |
| Leukaemia | 2,566 (5.1) | 4,472 (5.7) | 135 (1.2) | 96,478 (11.7) | 7,017 (3.8) | 22,229 (3.2) |
| Ovarian | 1,782 (3.5) | 2,871 (3.7) | 83 (0.7) | 32,791 (4.0) | 5,514 (3.0) | 20,631 (3.0) |
| Head and neck | 1,617 (3.2) | 2,826 (3.6) | 963 (8.5) | 25,771 (3.1) | 7,919 (4.3) | 30,781 (4.4) |
|  |  |  |  |  |  |  |
| Number of cases | 50,448 | 78,308 | 11,323 | 825,600 | 184,927 | 694,577 |

**Cancer classification (ICD-10):** C50: Breast; C18-C20: Colorectal; C67: Bladder; C61: Prostate; C82-C85 & C96: Non-Hodgkin lymphoma; C33-C34: Trachea, bronchus and lung; C88 & C90: Myeloma; C91-C95: Leukaemia C56: Ovarian; C00-C14 & C30-C32: Head and neck

**Supplementary material 3: Flowchart of study participants**

**CPES Sampling Frame**

109,475

| ***Ineligible*** | | **Eligible** | |
| --- | --- | --- | --- |
| ***Patients who died after sampling frame identification***  *6723* | ***Other ineligible patients (including those known to no longer be resident at recorded address and those who received the survey in error)***  *1431* | **Non-respondents**  34,058 | **Respondents**  67,613 |

**Patients with complete variable information (ethnicity and deprivation)**

100,234

| **Non-responders**  31,140 | **Respondents**  61,981 |
| --- | --- |

| **Patient death post-sampling**  5622 | **All other patients included in the sampling frame**  94,612 |
| --- | --- |

**Supplementary material 4: Relative frequency of cancer diagnoses among respondents (dark bars) and incident cases (white bars)**
